# Intraspecific variation in fine root morphology of European beech: a root order-based analysis of phenotypic root morphospace

**DOI:** 10.1007/s00442-024-05558-3

**Published:** 2024-05-02

**Authors:** Eva Rüther, Dietrich Hertel, Christoph Leuschner

**Affiliations:** Plant Ecology, University of Goettingen, Untere Karspüle 2, 37073 Göttingen, Germany

**Keywords:** *Fagus sylvatica*, Root plasticity, Specific root area, Soil chemistry, Soil depth

## Abstract

Fine roots are multifunctional organs that may change function with ageing or root branching events from primarily absorptive to resource transport and storage functions. It is not well understood, how fine root branching patterns and related root functional differentiation along the longitudinal root axis change with soil chemical and physical conditions. We examined the variation in fine root branching patterns (the relative frequency of 1st to 4th root orders) and root morphological and chemical traits of European beech trees with soil depth (topsoil vs. subsoil) and soil chemistry (five sites with acid to neutral/alkaline bedrock). Bedrock type and related soil chemistry had an only minor influence on branching patterns: base-poor, infertile sites showed no higher fine root branching than base-rich sites. The contribution of 1st-order root segments to total fine root length decreased at all sites from about 60% in the topsoil (including organic layer) to 45% in the lower subsoil. This change was associated with a decrease in specific root area and root *N* content and an increase in mean root diameter with soil depth, while root tissue density did not change consistently. We conclude that soil depth (which acts through soil physical and chemical drivers) influences the fine root branching patterns of beech much more than soil chemical variation across soil types. To examine whether changes in root function are indeed triggered by branching events or result from root ageing and diameter growth, spatially explicit root physiological and anatomical studies across root orders are needed.

**Supplementary Information:**

The online version contains supplementary material available at 10.1007/s00442-024-05558-3.

## Introduction

Fine roots play a key role in multiple plant functions, as they link the plant to the soil and its various resources (Fitter 2002). One of their functions is to absorb and transport nutrients and water, thereby contributing to biogeochemical cycles in ecosystems (Pregitzer 2002; Prieto et al. 2012; Bardgett et al. 2014; Freschet and Roumet 2017). Roots also influence the chemistry, texture and stability of soil and the process of pedogenesis (Daynes et al. 2013; Dignac et al. 2017). For better understanding the functioning of root systems, it is crucial to distinguish between root segments with different functions. In the past, a common approach in tree root studies was to assign roots to different diameter classes of e.g.,  < 2 or  < 1 mm, assuming that these “fine” and “finest roots” mainly serve resource uptake. However, tree roots  < 2 mm typically are heavily branched and consist of root segments of different morphology and probably variable functions (Fitter 1982). To address this variation, an alternative approach attempts to classify root segments according to their position in the root system’s branching hierarchy, proceeding from 1st-order roots near the distal root tip to higher orders in older segments through consecutive branching events (Pregitzer 2002; Guo et al. 2008; Ouimette et al. 2013). In the sense of Rose (1983), this is a developmental classification, with orders indicating the relative age of the root segments but not necessarily their function. In temperate forest trees, roots  < 2 mm typically consist of three to five root orders (Pregitzer et al. 2002; Kubisch et al. 2015). An advantage of this approach is that categorization based on orders may facilitate comparison among different species, which is often misleading when root diameters are used (Pregitzer et al. 2002).

While certain root features such as diameter, specific root surface area and root nutrient contents seem to change more gradually with increasing distance from the terminal root tip (Pregitzer et al. 2002; Kubisch et al. 2015), anatomical studies suggest that properties such as cortex thickness, the presence of secondary xylem, and the formation of a continuous cork layer as a protecting secondary peripheral tissue change more abruptly, possibly related to root branching (Guo et al. 2008). Axial variation in the latter traits thus suggests that root functions may be more closely related to root order than to root diameter. For example, Guo et al. (2008) and Long et al. (2013) observed in temperate tree species that 1st- through 3rd-order roots generally had little secondary development and were highly colonized by mycorrhizae, while 4th- and 5th-order roots generally lacked mycorrhizal colonization but showed the development of secondary xylem to support transport functions. Consequently, the shift from resource absorption to transport seems to occur in woody species in the 3rd or 4th root order (Robinson et al. 2003; Valenzuela-Estrada et al. 2008). This functional differentiation between different root orders is related to differences in longevity. First- and second-order roots tend to be younger and have a shorter lifespan than higher-order root segments (Wells et al. 2002; Guo et al. 2008).

The fine roots of a tree’s root system are exposed to considerable variation in physical and chemical conditions in the rooted soil volume, both in vertical and horizontal direction. From topsoil to subsoil, soil bulk density increases, while organic carbon (C) and nitrogen (N) contents and soil biological activity and related decomposition rate typically decrease (Jobbágy and Jackson 2000; Blume et al. 2010; Salomé et al. 2010). Roots forage for N and phosphorus (P) compounds preferably in the organic layer and mineral topsoil, where mineralization rate is high and soil bulk density low, and roots may face periodic exposure to drought (Hishi et al. 2006). Subsoils are usually poor in N and P and of higher bulk density, but may supply calcium (Ca) and magnesium (Mg) from bedrock weathering, and they are often the only source of water during rainless periods (Bache et al. 2008; Bolou-Bi et al. 2012; Uhlig et al. 2020; Sosa-Hernández et al. 2019; Querejeta et al. 2021; Pastore et al. 2022). Roots adapt to these variable environmental conditions by means of substantial morphological and physiological plasticity, conveyed through changes in root tissue density, xylem anatomy, peridermal structure, and the species composition of mycorrhizal fungi, and likely through modified branching patterns (Forde and Lorenzo 2001; McElrone et al. 2004; Ostonen et al. 2007). For example, the number of root tips per fine root mass, specific root length (SRL, length per root mass) and root N concentration are usually higher in the topsoil with abundant soil organic matter than deeper in the profile (Leuschner et al. 2004; Finér et al. 2007; Kirfel et al. 2019). Kubisch et al. (2015) observed a decreasing degree of fine root branching from the topsoil (0–10 cm) to deeper layers (20–30 cm) in six temperate broadleaf tree species. Further, it has been found that subsoil fine roots of a given diameter are on average older than topsoil roots (Kirfel et al. 2017).

Large variation in soil conditions is encountered by roots also when the same species is capable of colonizing acid and alkaline soils, as is the case in European beech (*Fagus sylvatica* L) and some temperate oak species (Leuschner et al. 2006; Leuschner and Ellenberg 2017). Not only soil acidity but also the availability of most plant nutrients and the activity of potentially toxic ions differ greatly between acid and alkaline soils, which might provoke adaptive physiological and morphological responses of the fine root system. For example, soil acidity and high Al^3+^ ion concentrations have been found to reduce fine root lifespan, while N deficiency may promote explorative root growth and fine root system expansion in acid soils (Richter et al. 2013; McCormack and Guo 2014; Jia et al. 2020). However, Leuschner et al. (2004) and Kirfel et al. (2019) reported only moderate variation in root morphological traits in a comparison of European beech forests on different bedrock types; yet, these studies did not investigate root branching patterns. In fact, the plasticity of tree root systems in response to variation in soil properties remains one of the least understood aspects of the biology of woody plants (Brunner et al. 2019; He et al. 2022).

We choose five European beech forests on largely different bedrock to investigate variation in fine root branching patterns and root morphology in dependence on soil chemical conditions (acid, base-poor to alkaline, base-rich soils) and soil depth (topsoil to subsoil), covering a large part of the soil chemical conditions encountered by beech in its Central European distribution range (Leuschner and Ellenberg 2017). Root segments were assigned to root orders according to the system proposed by Pregitzer et al. (2002), and important morphological and chemical traits (root diameter (MD), specific root length (SRL), specific root area (SRA), root tissue density (RTD), root N concentration) were analyzed for the different root orders.

We assumed that the architecture of the fine root system of European beech responds to both, different soil depths and variation in soil chemical conditions by adjusting the absorptive surface area of the root system, which should manifest in altered branching patterns and thus changes in the ratio of root orders in the fine root system. With the aim to characterize the phenotypic morphospace of beech fine roots realized in the range of soils colonized by the species, we tested the following hypotheses: (1) Root order has a marked influence on root trait variation across the fine root system, and (2) root trait heterogeneity (plasticity) is driven more by soil depth than bedrock type or soil acidity, with the prediction that the degree of branching (i.e., the number of 1st-order root segments on root strands with 2 mm maximum diameter) is markedly higher in the topsoil than subsoil, while trait differences between sites are smaller. Nevertheless, we hypothesized that (3) beech fine roots branch less in alkaline, N-richer soils than in acid, nutrient-poorer soils, as nutrient concentrations are higher. Finally, we hypothesized that (4) 1st- and 2nd-order roots in the subsoil have in response to higher soil bulk density and thus penetration impedance a larger diameter and tissue density and lower SRA than the corresponding root orders in the topsoil, while root N concentration is reduced due to impaired N supply in the subsoil.

## Materials and methods

### Study sites

The study was conducted in mature European beech forests on five different bedrock types ranging from highly acid, base-poor to neutral/alkaline, base-rich in northern-central Germany (Pleistocene sand: PleSan, Tertiary sand: TerSan, Triassic sandstone: TriSst, Quaternary loess: QuaLos, Tertiary basalt: TerBas). Since maximum distance between sites is 200 km, the climate (sub-oceanic cool-temperate) is broadly comparable at all sites (7.1–8.7 °C mean annual temperature, 709–902 mm mean annual precipitation; Deutscher Wetterdienst 2021, Climate Data Center). Four sites are located on Mesozoic and Tertiary bedrock in the low mountains near the city of Göttingen (central Germany) at colline to submontane elevation (TerSan, QuaLos, TriSst, TerBas), the fifth site is situated 150 km northwest of Göttingen in the Pleistocene lowlands west of Hannover (Grinderwald) on deposits of the penultimate Ice Age (Saalian) (PleSan). Due to the variation in bedrock types, the soils are dystric to eutric Cambisols with largely different soil textures (sandy to silty with elevated clay content) and base saturation (from  ~ 6 to 100% in the lower subsoil). All forests are monospecific mature beech forests with closed canopies and mean tree heights of 26–36 m, stem densities of 111 to 290 ha^−1^, tree ages of 100 to 153 years, mean diameters at breast height (DBH) of about 33–50 cm, and cumulative basal areas of 23 to 43 m^2^ ha^−1^ (Table 1). Forest communities belong to the Luzulo-Fagetum association on base-poorer substrates and to the Galio odorati-Fagetum on base-richer substrates. No other tree species than beech were present near the root sampling sites. Shrub layers were absent at all sites. A herb layer of *Galium odoratum* and other species with about 30–50% cover was present at the TerBas site, while the herb layer reached less than 10% cover at the other sites.

**Table 1:** Summary of stand characteristics and soil chemistry of the five investigated plots (see Kirfel et al. 2019)

		PleSan	TerSan	TriSst	QuaLos	TerBas
MAT (°C)		8.7	8.1	7.7	8.1	7.1
MAP (mm yr^−1^)		718	761	772	709	902
Bedrock type		Pleistocene glacio-fluviatile deposit	Tertiary sand	Triassic sandstone	Quaternary loess	Tertiary basalt
Soil type		DyCa	DyCa	DyCa	SeuCa	EuCa
Maximum profile depth (m)		≥ 2	≥ 2	0.6–0.8	≥ 2	0.6–0.8
Soil texture	Topsoil	Sandy loam	Sandy loam	Loam	Silt	Loam
	Upper subsoil	Loamy sand	Sandy loam	Silty loam	Silt	Silty loam
	Lower subsoil	Loamy sand	Sandy loam	Silty loam	Silt	Silty loam
Bulk soil density (g cm^−3^)	Topsoil	1.2	1.2	1.1	1.1	n.a
	Upper subsoil	1.5	1.4	1.5	1.4	n.a
	Lower subsoil	1.5	1.4	1.2	1.5	n.a
pH (CaCl_2_)	Topsoil	3.5	3.7	4.0	3.6	3.7
	Upper subsoil	4.1	4.1	3.9	3.7	4.1
	Lower subsoil	4.0	3.9	3.8	4.0	4.8
Base saturation (%)	Topsoil	9.0	4.8	26.8	16.3	14.2
	Upper subsoil	6.3	9.3	24.5	39.4	51.0
	Lower subsoil	6.2	10.7	22.7	99.5	97.5
Forest community		LF	LF	LF	GF	GF
Mean tree height (m)		26.8	35.3	36.1	32.9	29.1
Stem density (ha^−1^)		287	144	111	256	133
Tree age (years)		100	118	133	95	153
Mean DBH (cm)		33.1	45.2	46.7	40.2	50.2
Plot basal area (m^2^ ha^−1^)		27.1	24.6	22.8	37.3	43.2

Pleistocene sand: PleSan, Tertiary sand: TerSan, Triassic sandstone: TriSst, Quaternary loess: QuaLos, Tertiary basalt: TerBas. *MAT* mean annual temperature, *MAP* mean annual precipitation. Soil types: DyCa—Dystric Cambisol, SeuCa—Semi-eutric Cambisol, EuCa—Eutric Cambisol. Forest communities: LF—Luzulo-Fagetum, GF—Galio odorati-Fagetum

### Root sampling

Roots were collected in September and October 2017 in three different soil depths at the three sites with deep soil profiles (> 2 m depth; TerSan, PleSan, QuaLos), and at two depths at the two sites with more shallow profiles (up to 1 m depth; TriSst, TerBas). Soil layers were categorized as topsoil (0–20 cm), upper subsoil (20–110 cm) and lower subsoil (110–200 cm) based on detailed analyses of soil physical and chemical properties in the profiles (Heinze et al. 2018). The topsoil includes the organic layer on top of the mineral soil, which varied in depth from 19 to 44 mm. Three soil pits per site were dug at random positions in the stands, but with a minimum distance of 5 m to each other and maximum distance to adult beech trees of 2 m. At the three predefined soil depths (0–20 cm, 20–110 cm, 110–200 cm), each three fine root strands of ca 10 cm length and about 2 mm maximum root diameter were carefully excavated together with the adherent soil, resulting in nine replicate root strands per layer and site. The root strands comprised the intact terminal endings of the intensively branched fine root system, consisting of root segments assignable to 1st to 5th or higher orders. Root diameter at the proximal end of the strand was about 2 to 2.5 mm. In total, 18 root strands in the shallow soil profiles (topsoil and upper subsoil) and 27 roots in the deep profiles (topsoil, upper subsoil, lower subsoil) were analyzed, leading to 117 root strands in total. The roots with adherent soil were stored at 4 °C and further processed usually within 50 days (in a few cases up to 120 days).

### Assignment to root orders, and root morphological and chemical analyses

Only fine roots of European beech were considered in the analysis. While roots of other tree species were not present in the samples, roots of herbaceous plants accounted for a small proportion of root mass in the samples of the TerBas site, but were negligible at the other sites. Roots of herbaceous plants were easily separated from beech roots due to their much smaller root diameter; they were discarded. To analyze the morphology of the collected beech roots, all roots were gently washed with tap water to remove the adherent soil, following the procedure established by Meinen et al. (2009) and Kubisch et al. (2015). Intact root strands of around 10 cm length were scanned using a flat-bed scanner (EPSON expression 1680, EPSON America Inc., resolution 1600 × 3200 dpi) and WinRhizo 2005c software (Régent Instruments Inc., Quebec, Canada;) to analyze total root length, root surface area, root volume and root diameters.

After scanning, roots were dissected into the different root orders following the procedure outlined in Pregitzer et al. (2002). To do so, the most distal root segments (which typically form the mycorrhizal tip) to the first branching node were categorized as first root order, followed by segments assigned to the second to fifth order after further branchings. The root strands were dissected by order and the segments of an order pooled for further analysis.

The segments of a given root order were scanned again to measure order-specific root morphological traits, notably mean root diameter, total length, total surface area, and root volume, and the root mass was dried for 48 h at 70 °C and weighed. We then calculated for each soil depth and site the traits specific root length (SRL, in m g^−1^), specific root surface area (SRA, in cm^2^ g^−1^), and root tissue density (RTD, in g cm^−3^) for the 1st to 4th root order. In addition, the length and area of all segments of a given order in a strand was summed up and expressed in percent of the total root length and area of the strand (i.e., all root orders added). The degree of branching of the root strand was calculated as the proportion of the area (or length) of the first root order segments in the total root area (or length) of the root strand. The 1st order was chosen, as it contains the mycorrhizal root tips and is most likely the organ with highest resource uptake. The nitrogen and carbon contents of the different root orders were analyzed in the root dry mass by gas chromatography with an elemental analyzer (Vario EL, elementar, Hanau, Germany). Since tap water was used for root washing, a slight bias in the root N values cannot be excluded. To express the change in root traits of a given order from the topsoil to the subsoil in percent, we computed the difference of topsoil and subsoil values and related it to the topsoil value, which was set to 100%.

### Statistical analysis

All statistical analyses were conducted with R software (Version 4.0.2, R Core Team, Vienna, Austria). The distribution of the data was checked for normality with a Shapiro–Wilk test and QQ-plots. The data were tested for significant differences between different soil depths and root orders using one-way analysis of variance (ANOVA), followed by a Tukeys’s HSD test for pair-wise comparisons. A Kruskal–Wallis H-test followed by a Mann–Whitney *U*-test was performed, when the assumption of normal distribution was not met. To quantify relative trait differences between root orders and soil depths, we calculated trait means and standard errors for a given site, soil depth and root order. The resulting sample size was *n* = 9 for a given site, depth, and root order. To analyze the interaction between root traits, root orders and soil depths, we performed Principal Component Analyses (PCA) for the whole data set. Pearson correlation was employed to investigate relationships between root traits and between root traits and soil chemical and physical variables, based on means per soil layer and site. A significance level of *p* < 0.05 was used throughout the paper.

## Results

### Change in fine root branching with soil depth and its dependence on bedrock type

**Fig. 1:**
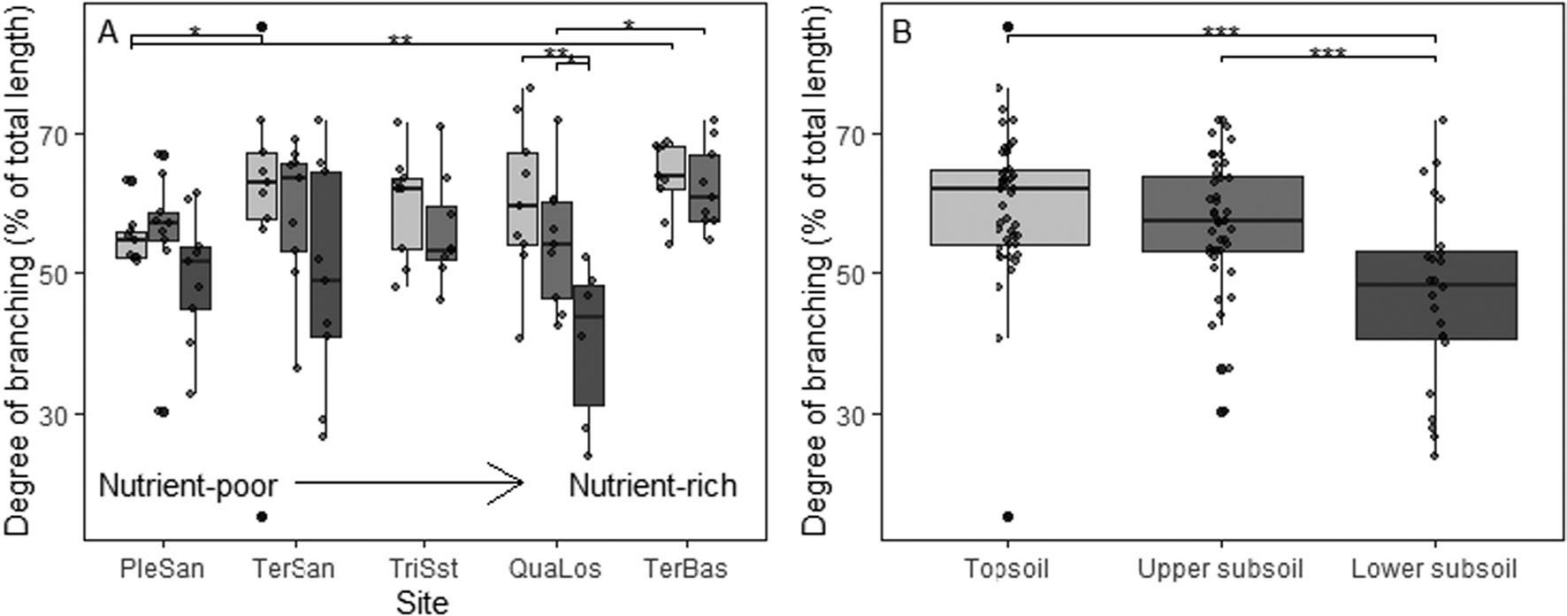
(**A**) Degree of root branching (length of 1st-order root segments in percent of total root length) in three soil depths at the five sites (PleSan: Pleistocene sand, QuaLos: Quaternary loess, TerBas: Tertiary basalt, TerSan: Tertiary sand, TriSst: Triassic sandstone) (box-whisker plots with median, 25- and 75-percentiles (box), and highest/lowest value not exceeding 1.5 inter-quartile ranges (whisker); *N* = 9). (**B**) Change in the degree of root branching (based on 1st-order length fraction) from the topsoil to the subsoil (all five sites pooled). Topsoil (0–20 cm), upper subsoil (20–110 cm) and lower subsoil (110–200 cm) (*N* = 45). Significant differences between sites for a given soil depth or between the three soil depths (with the five sites pooled) are indicated by horizontal lines and asterisks at the figure top (*p < 0.05, **p < 0.01, ***p < 0.001). *n* = 117

Forty to 64% of total root length (and 28–53% of root surface area) of the sampled fine root strands (maximum diameter ca. 2 mm) were contributed by first-order root segments, with the proportion varying with soil depth and among the five sites. The degree of branching, i.e., the proportion of length of the 1st-order segments in total root length, generally decreased from the topsoil (median of all sites: 60%) to the subsoil (45%; Fig. 1). However, differences between depths were significant at the site level only for the Quaternary Loess site (Mann–Whitney *U*-test, *n* = 18; upper subsoil vs. lower subsoil: *p* = 0.036, *R*^2^ = 0.34; topsoil vs. lower subsoil: *p* = 0.005, *R*^2^ = 0.61). Significant differences in branching degree between sites were found among the topsoils of the Pleistocene sand (PleSan), Tertiary sand (TerSan) and Tertiary basalt (TerBas) sites, with highest values observed at the TerBas site (64%) and lowest at the PleSan site (55%) (Mann–Whitney *U*-test, *n* = 18; topsoil of PleSan vs. TerSan *p* = 0.019, *R*^2^ = 0.31; topsoil of PleSan vs. TerBas *p* = 0.002, *R*^2^ = 0.50; upper subsoil of QuaLos vs. TerBas *p* = 0.04, *R*^2^ = 0.23). In the subsoil, a particularly low branching degree was found at the Quaternary loess (QuaLos) site (44%). Most fine root tips were found to be colonized by ectomycorrhizal fungi (typically  > 95%), independent of soil substrate and soil depth.

**Fig. 2:**
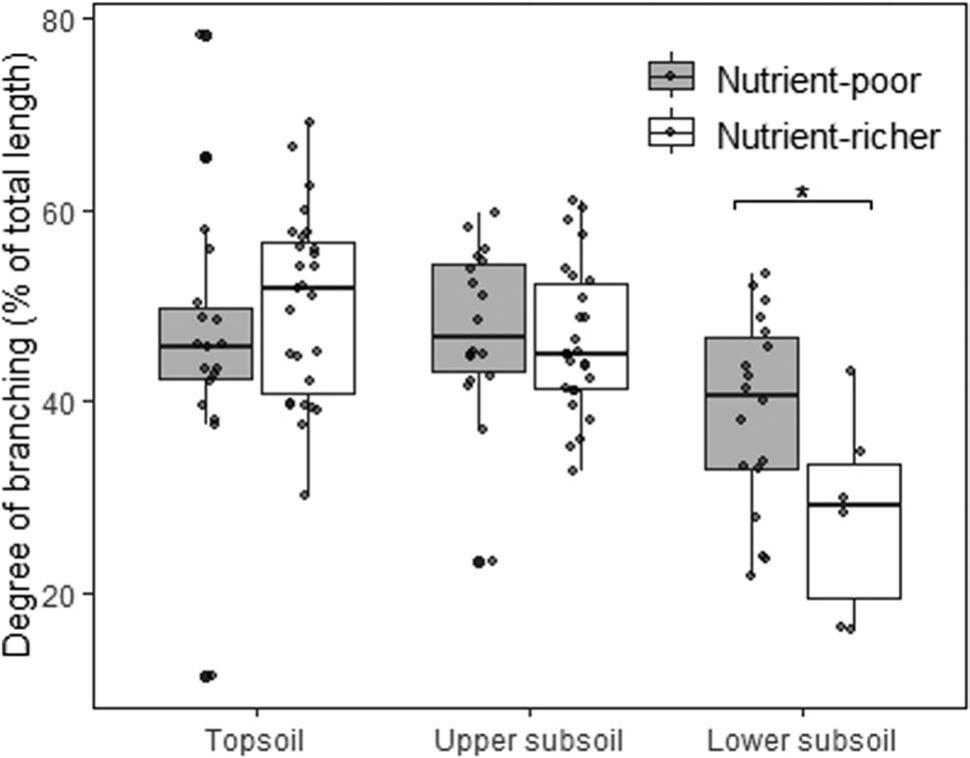
Degree of root branching (length of 1st-order root segments in percent of total root length) in topsoil, upper subsoil and lower subsoil at sandy and silty/clayey sites. Topsoil (0–20 cm), upper subsoil (20–110 cm) and lower subsoil (110–200 cm) (*N* = 18). Significant differences between the two groups in a given depth are indicated by horizontal lines and asterisks at the figure top (*p < 0.05). Sandy nutrient-poorer sites: Pleistocene sand, Tertiary sand, Triassic sandstone; silty/clayey nutrient-richer sites: Triassic basalt, Quaternary loess. *n* = 117

Pooling the nutrient-poorer sandy sites (PleSan, TerSan, TriSst) and the nutrient-richer silty/clayey sites (QuaLos, TerBas) shows no significant differences in the degree of branching between the two categories (Fig. 2).

### Root trait change across root orders

**Fig. 3:**
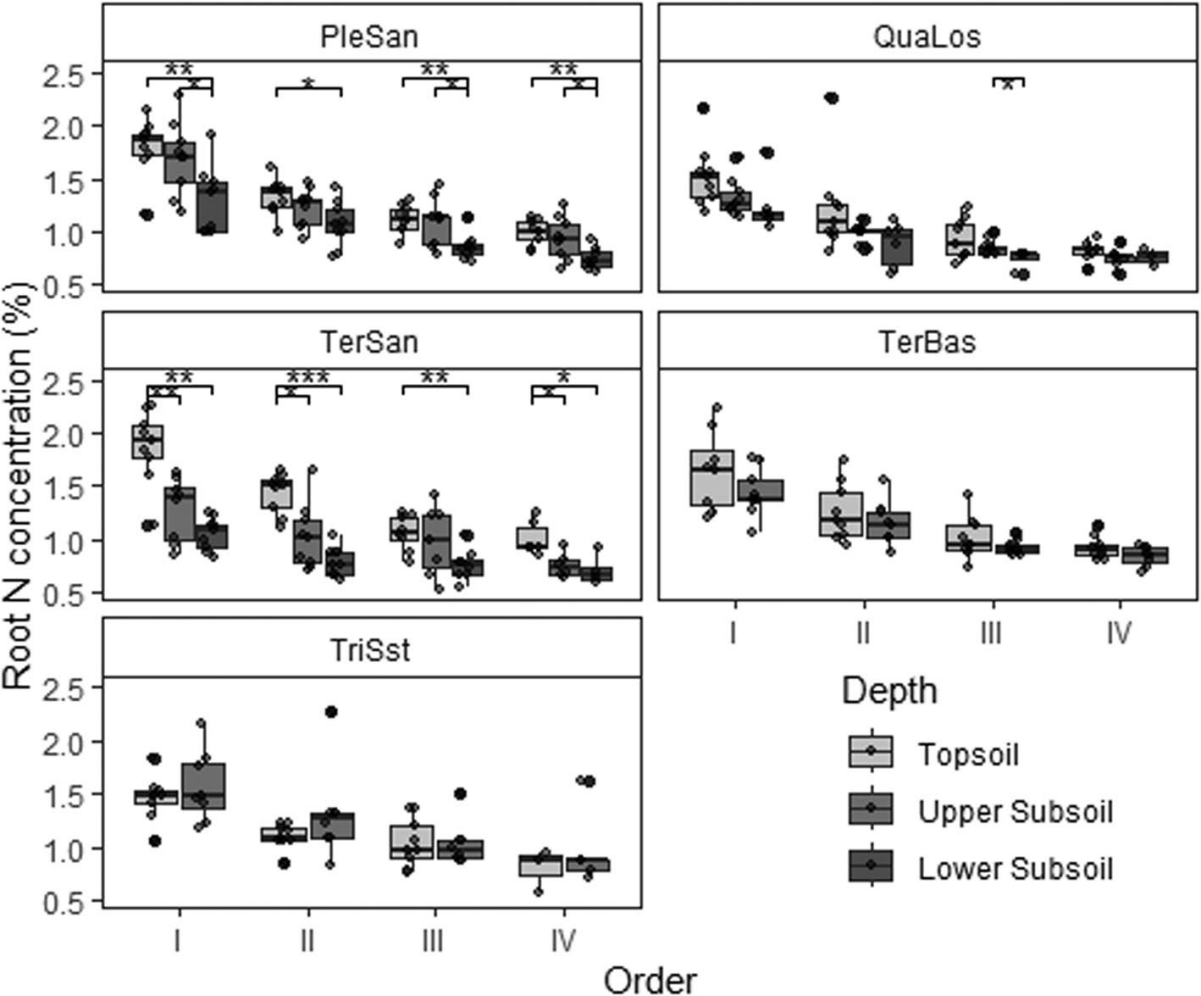
Root nitrogen concentration distribution in the first four root orders (I to IV) at the five sites in the three different soil depths (box-whisker plots with median, 25- and 75-percentiles (box), and highest/lowest value not exceeding 1.5 inter-quartile ranges (whisker); *N* = 9). Topsoil (0–20 cm), upper subsoil (20–110 cm) and lower subsoil (110–200 cm). PleSan: Pleistocene sand, QuaLos: Quaternary loess, TerBas: Tertiary basalt, TerSan: Tertiary sand, TriSst: Triassic sandstone. Significant differences between the soil depths at a site in a root order class are indicated by horizontal lines and asterisks at the figure top (*p < 0.05, **p < 0.01). For shallow sites: *n* = 72, for deeper sites *n* = 109

**Fig. 4:**
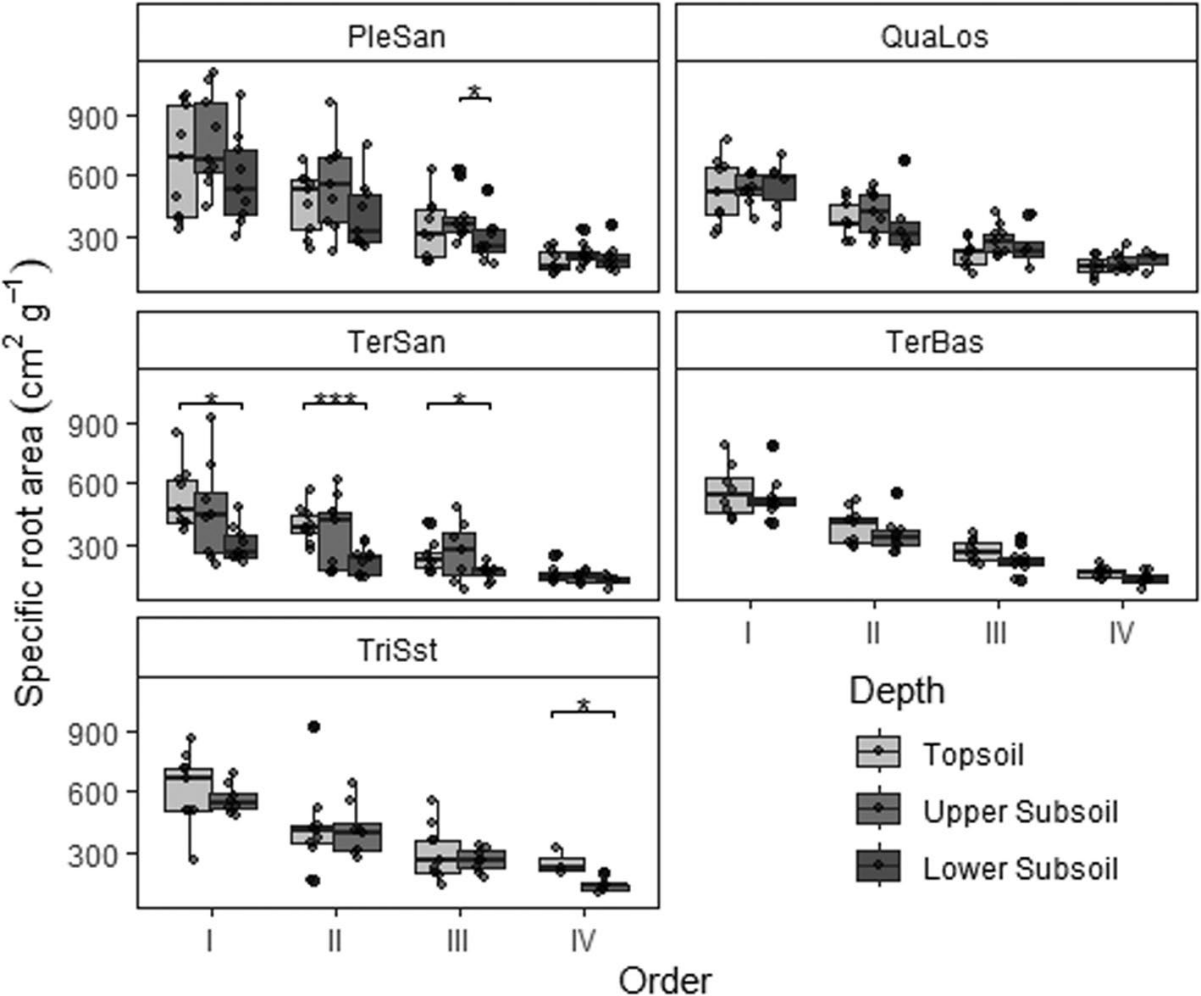
Specific root area distribution in the first four root orders (I to IV) at the five sites in the three different soil depths (box-whisker plots with median, 25- and 75-percentiles (box), and highest/lowest value not exceeding 1.5 inter-quartile ranges (whisker); *N* = 9). Topsoil (0–20 cm), upper subsoil (20–110 cm) and lower subsoil (110–200 cm)). PleSan: Pleistocene sand, QuaLos: Quaternary loess, TerBas: Tertiary basalt, TerSan: Tertiary sand, TriSst: Triassic sandstone. Significant differences between the soil depths at a site in a root order class are indicated by horizontal lines and asterisks at the figure top (*p < 0.05, **p < 0.01, ***p < 0.001). For shallow sites: *n* = 72, for deeper sites *n* = 109

**Fig. 5:**
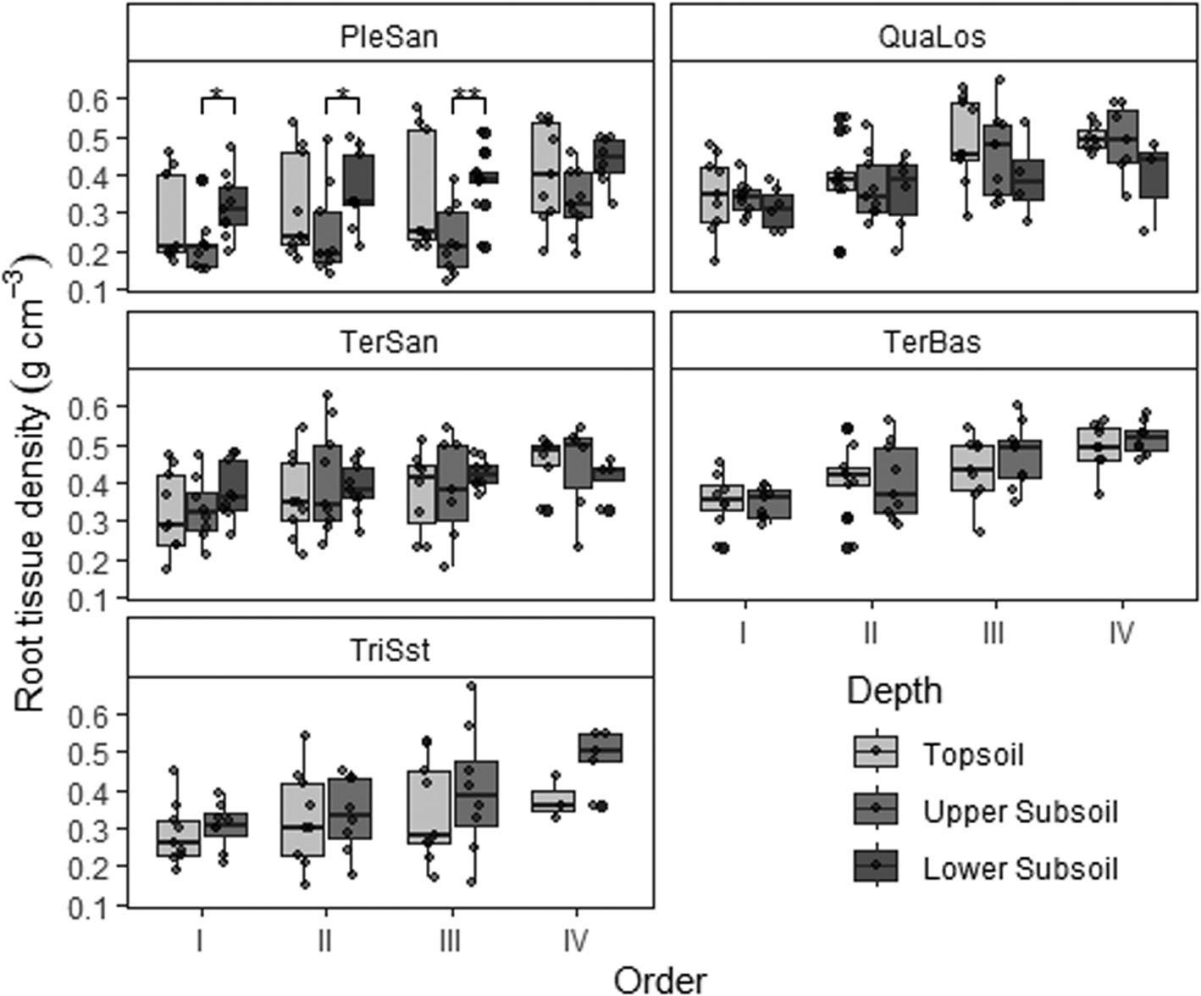
Root tissue density distribution in the first four root orders (I to IV) at the five sites in the three different soil depths (box-whisker plots with median, 25- and 75-percentiles (box), and highest/lowest value not exceeding 1.5 inter-quartile ranges (whisker); *N* = 9). Topsoil (0–20 cm), upper subsoil (20–110 cm) and lower subsoil (110–200 cm). PleSan: Pleistocene sand, QuaLos: Quaternary loess, TerBas: Tertiary basalt, TerSan: Tertiary sand, TriSst: Triassic sandstone. Significant differences between the soil depths at a site in a root order class are indicated by horizontal lines and asterisks at the figure top (*p < 0.05, **p < 0.01). For shallow sites: *n* = 72, for deeper sites *n* = 109

We found a consistent decrease in root N concentration (Fig. 3) and specific root area (SRA, Fig. 4) from the first to the fourth root order across the five sites; a similar pattern was observed for specific root length (SRL; data not shown). The proportion of root area (and root length) that is contributed by a given order to total root length and area also decreased consistently from the 1st to the 4th order (Fig. S1 in the Supplement). In contrast, root tissue density (RTD, Fig. 5) and root diameter (Fig. S2 in the Supplement) increased from the first to the fourth order. The change in root traits from 1st- to 4th-order segments was broadly similar in the three soil depths and also among the five sites on different bedrock. However, the roots in the lower subsoil of the nutrient-poorest PleSan site were for a given root order thinner, but with higher tissue density and lower SRA and root N content than corresponding roots in topsoil and upper subsoil. In contrast, the roots in the lower subsoil of the TerSan site were thicker with smaller SRA than corresponding root orders higher up in the profile, while RTD and root N content differed only slightly.

### Root trait change with soil depth

To compare the traits of 1st-order root segments of topsoil roots with subsoil roots (lower subsoil in case of the three deep profiles and upper subsoil for the two more shallow profiles), the relative difference in values between the two depths was calculated (Table 2). SRA of 1st- and 2nd-order segments decreased from the topsoil to the subsoil by 5–43% (except for a slight downward decrease of SRA in the 1st-order category at the QuaLos site). For mean root diameter of the 1st- and 2nd-order class, we found increases by 3–71% from top- to subsoil at all sites (except for the TriSst site with a slight downward diameter decrease by 3–9%; Table 2). Root N concentration in the first two root orders decreased by 9–45% from the top- to the subsoil (with the exception of the TriSst site with an increase by 7–19%). We found no consistent change in the RTD of 1st- and 2nd-order roots with soil depth at the five sites. At the three sandy sites, RTD increased by around 2–12% from the top- to the subsoil, while it decreased at the silty/clayey sites. The TerSan site was the location where the largest number of trait differences between topsoil and subsoil of 1st- and 2nd-order roots were significant. At the other sites, the majority of vertical trait differences were not significant (Figs. 3, 4 and 5).

**Table 2:** Relative difference (%) in root traits between topsoil and subsoil (upper subsoil in case of shallow profiles, lower subsoil in case of deep profiles) for 1st-order (I) and 2nd-order root segments (II) at the five sites

Plot	Order	SRA	*D*	RTD	N
PleSan	I	− 12.8	**2.8**	**7.9**	− 27.7
	II	− 15.2	**4.2**	**12.6**	− 19.2
TerSan	I	− 43.2	**47.8**	**16.0**	− 44.0
	II	− 43.8	**70.5**	**6.8**	− 44.5
TriSst	I	− 8.2	− 3.0	**6.8**	**7.2**
	II	− 5.2	− 9.1	**2.6**	**18.5**
QuaLos	I	**4.3**	**4.4**	− 11.0	− 18.5
	II	− 5.9	**23.9**	− 10.3	− 27.7
TerBas	I	− 5.7	**5.2**	− 0.6	− 13.5
	II	− 11.1	**13.8**	− 1.1	− 9.0

Numbers give subsoil mean—topsoil mean/topsoil mean; positive values are printed in bold. SRA (specific root area), SRL (specific root length), *D* (root diameter), RTD (root tissue density), N (root N concentration)

### Dependence of root traits on soil chemical and physical factors

A Pearson correlation table between root traits and soil variables revealed a dependence of root traits neither on soil pH, base saturation or soil C/N ratio, nor on bulk soil density. An exception is the positive relation between root N concentration and soil C/N ratio (Table 3). As expected, SRA was positively related to root N and negatively to RTD and root diameter.

**Table 3:** Pearson correlation between different traits of first-order root segments (root diameter, specific root area (SRA) and root tissue density (RTD)) and soil chemical and physical variables (C/N ratio, base saturation, pH and bulk soil density) across the five sites (based on means of the different soil layers and sites)

	SRA	RTD	Root N	Soil C/N	Base saturation	Soil pH	Bulk soil density
Root diameter	** − 0.598 (0.031)**	0.162 (0.597)	* − 0.499 (0.083)*	− 0.231 (0.447)	− 0.189 (0.536)	0.29 (0.336)	0.187 (0.582)
SRA		** − 0.859 (< 0.001)**	**0.633 (0.020)**	0.446 (0.126)	− 0.106 (0.729)	0.022 (0.943)	0 (1.0)
RTD			− 0.429 (0.143)	− 0.291 (0.335)	0.161 (0.599)	− 0.316 (0.292)	− 0.178 (0.600)
Root N				**0.776 (0.002)**	− 0.396 (0.180)	− 0.435 (0.137)	− 0.408 (0.213)
Soil C/N					* − 0.544 (0.054)*	− 0.443 (0.130)	** − 0.607 (0.048)**
Base saturation						0.19 (0.533)	0.32 (0.337)
Soil pH							*0.588 (0.057)*

Given are *R*^2^ values and *p* values in brackets. Soil data after Kirfel et al. (2019). Significant relationships are printed in bold, marginally significant ones (0.1 > *p* > 0.05) in italics

**Fig. 6:**
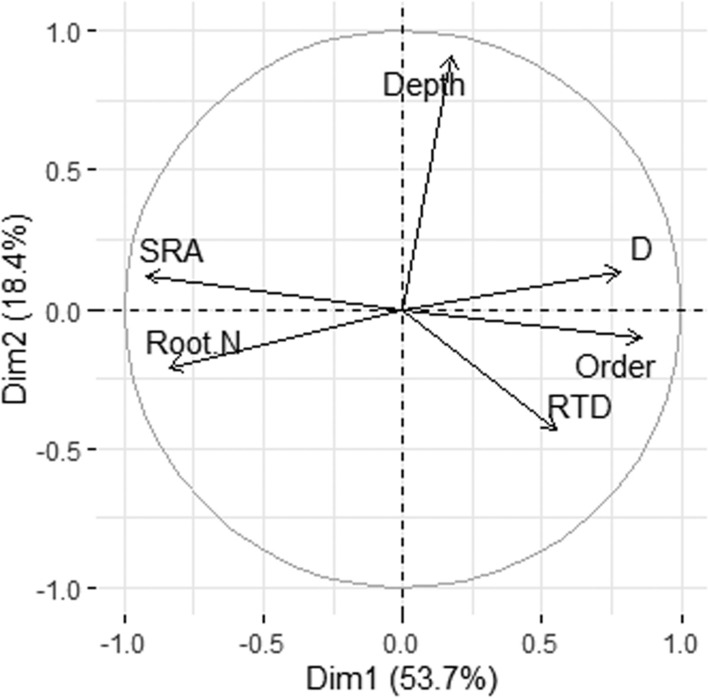
PCA conducted with all root morphological and chemical traits (SRA, root diameter *D*, root tissue density RTD, root N concentration) and root order (Order) and soil depth (Depth) in the whole data set (all five sites)

To examine the relative importance of root order for root trait variation, a PCA was run on the interaction between the four major studied root morphological and chemical traits, and root order and soil depth (Table 4, Fig. 6). Root order was closely related to the first PCA axis and correlated with all morphological and chemical traits, pointing at a considerable influence of root branching on root traits. Root order showed the closest association with SRA (negative), followed by root N concentration (negative) and root diameter (positive), while the relation to RTD was weak. Soil depth was the most important factor correlating with the second axis (Fig. 6, Table 4); its relation to root traits was fairly weak, however. This is also valid when conducting the PCA separately for the five sites (Table S1).

**Table 4:** Results of a PCA conducted with all root morphological and chemical traits and root order and soil depth in the whole data set (all five sites)

	Axis 1	Axis 2
Eigenvalues	3.224	1.1
Depth	0.177	0.910
Root order	0.855	− 0.104
Root diameter	0.779	0.135
SRA	− 0.921	0.123
RTD	0.552	− 0.431
Root N	− 0.838	− 0.207

Values give loadings of displayed traits on the PCA axes. Corresponding PCAs were run for the different sites separately (Table S1 in the Supplement)

## Discussion

Root order was identified as a major factor influencing the traits of beech fine roots, as is visible in the figures on trait variation across orders and also reflected by the PCA (hypothesis 1). Our results also strongly support hypothesis (2) that branching intensity varies more in response to soil depth than with variation in mineralogy or soil acidity, but we found no consistent evidence for a higher root branching in less fertile, N-poorer soils (hypothesis 3). In support of hypothesis (4), subsoil roots were on average thicker with lower SRA and N content.

### Root plasticity is driven more by soil depth than soil mineralogy or acidity

Our analysis of intraspecific variation in fine root system morphology of beech shows in support of hypothesis (2) that the degree of branching, here measured as the length of 1st-order root segments in percent of total fine root length, varies considerably from topsoil to subsoil, while differences among the five sites on different bedrock are unexpectedly small. In correspondence, the morphological root traits varied independently of soil pH and base saturation. At most sampling locations, about 40–55% of total root length of the sampled fine root strands of ca. 10 cm length were contributed by 1st-order segments, which are almost exclusively assignable to root tips colonized by different types of ectomycorrhizae. The remainder referred to 2nd- to 4th-order root segments. In the pooled sample of all five sites, the degree of branching decreased from the topsoil (0–20 cm, which includes a 2 to 4 cm thick ectorganic layer) to the subsoil (110–200 cm) from ca 60 to 45%, with only slight differences existing between 0–20 cm and 20–110 cm depth. Thus, 2nd- and especially 3rd- and 4th-order roots constitute a greater fraction of the fine root system in the subsoil than in the topsoil. A likely explanation is that the typically elongated, sparsely branched tap roots in the subsoil primarily have a transport function, whereas the highly branched topsoil roots are mainly serving for resource uptake. The largest change in fine root morphology and branching patterns is expected to occur in the few centimeters between the organic layer and the mineral topsoil, with highest SRL, SRA and root N content and a higher branching intensity found in the former (Kirfel et al. 2019). This shift in root morphology is obscured in our data, as we bulked the organic layer with the uppermost mineral soil material in our topsoil samples.

Our results can be compared to only few other studies in mature temperate forests, in which changes in root branching in the profile down to the subsoil have been studied. Our data match findings from a mixed beech forest on limestone, where branching intensity (measured as the number of fine root tips per root mass) more than halved in the profile from 0–10 to 20–30 cm depth (Kubisch et al. 2015). Reduced branching at greater soil depth is associated with morphological and chemical changes in the root orders themselves, notably in the youngest, most distal orders. At most sites, 1st- and 2nd-order roots were in the lower subsoil somewhat thicker with lower SRA and lower N concentration than in the topsoil, which supports our hypothesis (4). This could be a response to higher soil bulk density (Alameda and Villar 2012; Freschet et al. 2020), as soil bulk density increased by about 0.3 g cm^−3^ from the upper to the lower mineral soil in our profiles. Thus, surface reduction toward deeper soil horizons involves both a reduction in the area fraction of 1st- and 2nd-order roots and a decrease in the surface development of the roots in these orders. In contrast, RTD showed no consistent change with increasing soil depth in 1st- and 2nd-order roots (increase at three sites, decrease at two sites). This supports the notion that root tissue density may vary independently of SRA and SRL (Kramer-Walter et al. 2016) and that plants can achieve a surface reduction in their fine root system regardless of whether RTD is increased or decreased.

Both anatomical studies and stable isotope data from tree root systems suggest that nutrient and water uptake are primarily taking place in 1st- and 2nd-order roots, while 4th- and higher-order root segments predominantly serve for water and nutrient transport and possibly storage (Pregitzer et al. 2002; Robinson et al. 2003; Xia et al. 2010; Long et al. 2013; Adams and Eissenstat 2014; McCormack et al. 2015). According to this understanding of root functional differentiation, 3rd-order root segments may be considered as a transition zone between uptake and transport function (Valenzuela-Estrada et al. 2008). However, this concept must remain hypothetical with respect to beech roots, until measurements can quantify the uptake and transport activities of the different root orders. Yet, the typical habit of beech roots in the subsoil with an extended main axis built by 3rd- and 4th-order roots and only few 1st- and 2nd-order roots suggests that water conduction is a main function of these roots, while nutrient uptake, which presumably scales with the number of mycorrhizal tips and thus 1st-order roots, is probably less important in the subsoil. As root water uptake in drying soil depends largely on hydraulic resistances in the rhizosphere, root cortex and endodermis (Carminati and Javaux 2020), and plants are able to enhance it by increasing the soil-to-root water potential gradient, we speculate that reducing the proportion of 1st- and 2nd-order roots in the subsoil will reduce nutrient uptake more than water uptake. This matches the widely observed vertical decrease in soil N content, soil biological activity and N mineralization rate in forest soils (Runge 1983; Leuschner and Ellenberg 2017), which increases the cost/benefit ratio of operating roots for the primary goal of nutrient uptake in the subsoil. At three of the five sites, subsoil roots had lower root N concentrations than topsoil roots in a given root order, suggesting increasing N shortage, which might limit root growth in the subsoil.

Our site comparison shows only an inconsistent effect of soil chemistry and nutrient availability on root branching intensity, matching earlier results that showed only minor SRA change across our five sites (Kirfel et al. 2019). This supports our second hypothesis, but does not confirm the third hypothesis that postulated higher branching at sites with lower nutrient availability. While branching intensity was somewhat (but not significantly) higher in the subsoil of the nutrient-poorer sandy sites than at the richer loamy/clayey sites, no difference was visible higher up in the profile, and branching in the topsoil tended to be even higher at the richer sites. This contradicts the notion that plants exposed to nutrient shortage should produce finer, more branched roots with greater surface area to increase resource uptake and improve the cost/benefit ratio of root formation and maintenance (Pregitzer et al. 2002; Ostonen et al. 2007). In fact, Kramer-Walter et al. (2016) studied 66 tree species in New Zealand and found higher branching intensity at the more infertile sites. However, the concept of nutrient scarcity as a driver of root branching was developed primarily to address interspecific trait differences and may require modification, when applied to intraspecific root trait variation. In support of our findings, a study in another six beech forest soils in Germany with variable soil chemistry also found only small site differences in fine root morphology and no consistent relation between soil chemistry and SRA (Leuschner et al. 2004). In our study, the only significant soil chemistry effect on root traits was the positive relation between root N concentration and soil C/N ratio, which comes as a surprise. However, this relation is plausible when considering that soil C/N ratio decreases with soil depth in the studied profiles (Heinze et al. 2018), and higher N concentrations in topsoil roots are related to higher soil C/N ratios, but also elevated mass-specific N concentrations, and higher N mineralization rates in comparison to the subsoil. Moreover, root N contents might well reflect root physiological activity (which presumably is higher in the topsoil) rather than soil N availability.

We cannot exclude that other environmental factors, notably P availability, ECM fungal species composition and water availability, are additional important drivers of branching intensity to those factors investigated here (soil pH and C/N ratio, base saturation). In any case, the variation in root branching patterns of beech in response to soil chemical gradients is remarkably small, given the range of bedrock types covered.

### Change in root traits with root order

The continuous decrease in root tissue N concentration with increasing root order is a reflection of decreasing metabolic activity and may be explained by reduced nutrient and water uptake rates in higher orders (Pregitzer et al 1998; Pregitzer et al. 2002; Guo et al. 2004; Fan and Jiang 2010), since it has been found that about 65% of root respiration may be attributable to root growth and ion uptake (*Quercus suber*, van den Boogaard in Lambers et al. 1982).

Root diameter and RTD showed a continuous increase, and SRL and SRA a continuous decrease, from the 1st to the 4th order at all study sites and all investigated soil depths. Similarly, Pregitzer et al. (2002) recorded no abrupt change in root traits between the first three root orders in nine North American temperate tree species. The lack of a step change in diameter and other traits from the 2nd to the 3rd and from the 3rd to the 4th order is interesting, as it is thought that the transition from absorptive to transport function should happen in this development stage (Valenzuela-Estrada et al. 2008; Long et al. 2013). The transition from root primary to secondary growth begins, when the procambium meristem between xylem and phloem becomes the circular vascular cambium meristem, and the pericycle develops into the cork cambium (phellogen), which produces the peridermis, a suberized cortical layer that prevents the entry of water. In beech, absorptive roots are assumed to consist of root tips colonized by mycorrhizae with a characteristic Hartig net (1st-order roots) and adjacent 1st-order and probably also 2nd-order root segments that are still in the stage of primary growth, i.e., possess an intact endodermis, cortex and rhizodermis. In our study, 1st-order roots of beech had diameters of 0.20–0.30 mm and 2nd-order roots of 0.25–0.40 mm, with somewhat larger diameters in the subsoil, which may be a response to higher soil bulk densities. With the transition to secondary growth, the secondary xylem widens and mean conduit diameters of beech roots increase, enhancing the hydraulic efficiency of the ageing root and its transport function (Kirfel et al. 2017). Since traits changed continuously from the 1st- to higher-order roots, it is unlikely that branching events are a trigger of root trait change along the longitudinal root axis, but the change is presumably a consequence of the shift from primary to secondary growth with root ageing and the associated root anatomical changes. A positive relationship between root age and diameter has been found in many studies (Guo et al. 2008; McCormack et al. 2012; Kirfel et al. 2017), but it is not clear, how the increase in root girth varies with root order. The 3rd- and 4th-order roots of our study must have been several years old, as the investigated fine root strands had at sections with 2 mm diameter median ages of about 10–16 years according to annual ring counting (Rüther et al. unpubl. results). We thus assume that the shift in root functions occurs gradually in beech fine roots and probably is more closely related to the degree of periderm development than to branching order itself. We postulate that root orders exert their influence on root functionality primarily through effects on root longevity, in that 1st- and 2nd-order roots are shed earlier upon stress than higher-order roots, which have a higher survival rate under unfavorable conditions. This is visible in scars left on the main root branches from shed 1st- and 2nd-order roots (Pregitzer et al. 2002). The regrowth of 1st- and 2nd-order roots results in young root tissue with initially primary growth and a cortex that is transmissive for water and nutrients and susceptible to colonization by mycorrhizal hyphae. If longitudinal root growth stagnates and the transition to secondary growth impedes further nutrient and water uptake and the root segments become uneconomic, 1st- and 2nd-order roots are probably shed because of age. In case of progressing longitudinal root growth, however, 1st- and 2nd-order roots mature to higher-order roots with transport function in the natural process of root ageing and associated secondary growth.

## Conclusion

Our analysis of the fine root morphology of European beech across a broad soil chemical gradient is one of the very few studies that explores the root phenotypic morphospace of a tree species with broad tolerance of soil chemical conditions. Root order was identified as a major determinant of root traits. Contrary to expectation, the response of root branching patterns of beech roots to a pronounced soil chemistry gradient was small. A more pronounced change in the degree of branching was detected between topsoil and subsoil, with 3rd- and 4th-order roots constituting a larger fraction in the subsoil than in the topsoil. The lower proportion of 1st-order root segments in the subsoil may suggest that water transport is a major function of these roots, while the uptake of N and other macronutrients may be less important. The SRA decrease from top- to subsoil was not consistently related to a RTD increase, suggesting that root surface reduction can be achieved independently of changes in root tissue density. The absence of marked step changes in root morphology and chemistry from the 2nd- to higher root orders may indicate a more gradual change in root function along the root axis. Spatially resolved root physiological and anatomical studies across different root orders are needed to clarify, whether root function shifts abruptly from 2nd- or 3rd-order to higher order roots, as is generally assumed, or changes more gradually across orders.

### Supplementary Information

Below is the link to the electronic supplementary material.

Supplementary file1 (DOCX 55 KB)

## Data Availability

The data are available from the authors upon reasonable request.
