# Supplementary material for: Intraspecific variation in fine root morphology of European beech: a root order-based analysis of phenotypic root morphospace
Source: Oecologia. 2024 May 2;205(1):121–33. doi: 10.1007/s00442-024-05558-3 (PMC11144161; doi:10.1007/s00442-024-05558-3)
Supplement: Supplementary file 1 — Supplementary file1 (DOCX 55 KB) [file 442_2024_5558_MOESM1_ESM.docx]

Electronic **Supplement Material**

Fig. S1. Contribution of the 1^st^ to 4^th^ root order to total root length (A) and total root area (B) in percent of root length in the whole root strand investigated (box-whisker plots with median, 25- and 75-percentiles (box), and highest/lowest value not exceeding 1.5 inter-quartile ranges (whisker)). n=117.

Fig. S2. Root diameter distribution in the first four root orders (I to IV) at the five sites in the three different soil depths (box-whisker plots with median, 25- and 75-percentiles (box), and highest/lowest value not exceeding 1.5 inter-quartile ranges (whisker)). Topsoil (0-20cm), upper subsoil (20-110cm) and lower subsoil (110-200cm). PleSan: Pleistocene sand, QuaLos: Quaternary loess, TerBas: Tertiary basalt, TerSan: Tertiary sand, TriSst: Triassic sandstone. Significant differences between the soil depths at a site in a root order class are indicated by horizontal lines and asterisks at the figure top (* p<0.05, ** p<0.01, *** p<0.001). For shallow sites: n=72, for deeper sites n=109.


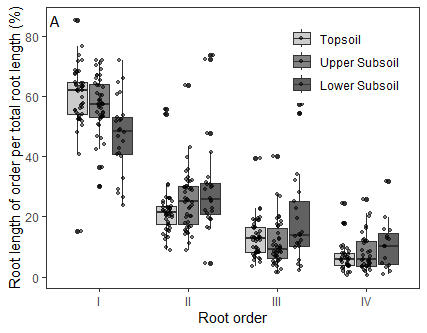

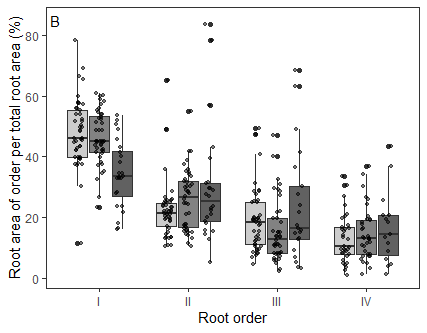


Figure S1


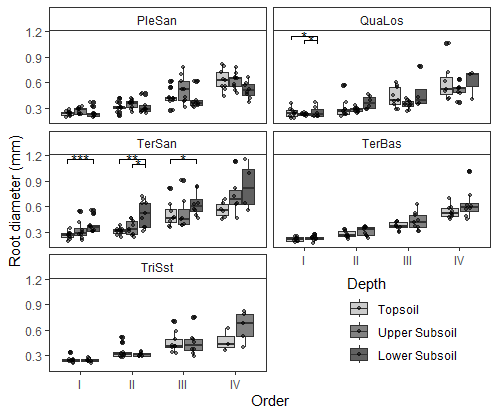


Figure S2

**Table S1.** Results of PCAs with inclusion of all root morphological and chemical traits and root order and soil depth, conducted separately for the five sites. Values give loadings of displayed traits on the PCA axes.

| **PleSan** | **Axis 1** | **Axis 2** | **QuaLos** | **Axis 1** | **Axis 2** |
| --- | --- | --- | --- | --- | --- |
| Eigenvalues | 3.372 | 1.244 | Eigenvalues | 3.409 | 1.19 |
| Depth | 0.151 | 0.782 | Depth | 0.059 | -0.960 |
| Root order | 0.874 | -0.311 | Root order | -0.916 | -0.045 |
| Root diameter | 0.743 | -0.520 | Root diameter | -0.750 | -0.081 |
| SRA | -0.936 | -0.040 | SRA | 0.948 | -0.086 |
| RTD | 0.625 | 0.445 | RTD | -0.728 | 0.338 |
| Root N | -0.876 | -0.256 | Root N | 0.758 | 0.372 |
| **TerSan** | **Axis 1** | **Axis 2** | **TerBas** | **Axis 1** | **Axis 2** |
| Eigenvalues | 3.209 | 1.208 | Eigenvalues | 3.879 | 1.017 |
| Depth | 0.428 | 0.783 | Depth | 0.133 | 0.988 |
| Root order | 0.746 | -0.347 | Root order | 0.932 | -0.156 |
| Root diameter | 0.839 | 0.117 | Root diameter | 0.841 | 0.022 |
| SRA | -0.919 | 0.139 | SRA | -0.961 | 0.043 |
| RTD | 0.342 | -0.637 | RTD | 0.794 | -0.061 |
| Root N | -0.897 | -0.190 | Root N | -0.855 | -0.099 |
| **TriSst** | **Axis 1** | **Axis 2** |  |  |  |
| Eigenvalues | 3.309 | 1.113 |  |  |  |
| Depth | 0.142 | 0.905 |  |  |  |
| Root order | 0.880 | -0.144 |  |  |  |
| Root diameter | 0.762 | -0.184 |  |  |  |
| SRA | -0.933 | -0.129 |  |  |  |
| RTD | 0.685 | 0.364 |  |  |  |
| Root N | -0.772 | 0.300 |  |  |  |

**Table S2.** Pearson correlation between different traits of second-order root segments (root diameter, specific root area (SRA) and root tissue density (RTD)) and soil chemical and physical variables (C/N ratio, base saturation, pH and bulk soil density) across the five sites (based on means of the different soil layers and sites). Given are R^2^ values and p values in brackets. Soil data after Kirfel et al. (2019). Significant relationships printed in bold, marginally significant ones (0.1 > p > 0.05) in italics.

|  | SRA | RTD | Root N | Soil C/N | Base Saturation | Soil pH | Bulk soil density |
| --- | --- | --- | --- | --- | --- | --- | --- |
| Root Diameter | **-0.573 (0.041)** | -0.023 (0.94) | **-0.654 (0.015)** | -0.31 (0.302) | 0.007  (0.982) | 0.354 (0.235) | 0.233  (0.490) |
| SRA |  | **-0.775 (0.002)** | **0.569 (0.042)** | 0.444 (0.129) | -0.213  (0.484) | -0.081 (0.792) | -0.032 (0.924) |
| RTD |  |  | -0.258 (0.394) | -0.3  (0.319) | 0.186  (0.542) | -0.128 (0.677) | -0.178  (0.600) |
| Root N |  |  |  | **0.721 (0.005)** | -0.468  (0.107) | -0.421 (0.152) | -0.384 (0.244) |
| Soil C/N |  |  |  |  | *-0.544*  *(0.054)* | -0.443 (0.130) | **-0.607 (0.048)** |
| Base Saturation |  |  |  |  |  | 0.19 (0.533) | 0.32  (0.337) |
| Soil pH |  |  |  |  |  |  | *0.588*  *(0.057)* |
